# Enhancement of Thermostability of *Aspergillus flavus* Urate Oxidase by Immobilization on the Ni-Based Magnetic Metal–Organic Framework

**DOI:** 10.3390/nano11071759

**Published:** 2021-07-06

**Authors:** Neda Motamedi, Mahmood Barani, Azadeh Lohrasbi-Nejad, Mojtaba Mortazavi, Ali Riahi-Medvar, Rajender S. Varma, Masoud Torkzadeh-Mahani

**Affiliations:** 1 Department of Biotechnology, Institute of Science, High Technology & Environmental Sciences, Graduate University of Advanced Technology, Kerman 7631133131, Iran; neda.m2950@yahoo.com (N.M.); mortezavimm@gmail.com (M.M.); 2 Medical Mycology and Bacteriology Research Center, Kerman University of Medical Sciences, Kerman 7616913555, Iran; mahmoodbarani7@gmail.com; 3 Department of Agricultural Biotechnology, Shahid Bahonar University of Kerman, Kerman 7616914111, Iran; azilohrasbi@gmail.com; 4 Department of Molecular and Cell Biology, Faculty of Basic Sciences, Kosar University of Bojnord, Bojnord 9415615458, Iran; riahi.ali@gmail.com; 5 Regional Centre of Advanced Technologies and Materials, Czech Advanced Technology and Research Institute, Palacký University in Olomouc, Šlechtitelů 27, 783 71 Olomouc, Czech Republic

**Keywords:** urate oxidase, magnetic metal–organic frameworks, immobilization, thermal stability

## Abstract

The improvement in the enzyme activity of *Aspergillus flavus* urate oxidase (Uox) was attained by immobilizing it on the surface of a Ni-based magnetic metal–organic framework (NimMOF) nanomaterial; physicochemical properties of NimMOF and its application as an enzyme stabilizing support were evaluated, which revealed a significant improvement in its stability upon immobilization on NimMOF (Uox@NimMOF). It was affirmed that while the free Uox enzyme lost almost all of its activity at ~40–45 °C, the immobilized Uox@NimMOF retained around 60% of its original activity, even retaining significant activity at 70 °C. The activation energy (Ea) of the enzyme was calculated to be ~58.81 kJ mol^−1^ after stabilization, which is approximately half of the naked Uox enzyme. Furthermore, the external spectroscopy showed that the MOF nanomaterials can be coated by hydrophobic areas of the Uox enzyme, and the immobilized enzyme was active over a broad range of pH and temperatures, which bodes well for the thermal and long-term stability of the immobilized Uox on NimMOF.

## 1. Introduction

Urate oxidase (Uox; EC 1.7.3.3), also identified as uricase, catalyzes the uric acid oxidation to allantoin and is a significant enzyme with several therapeutic and diagnostic applications [1]. In biological fluids, such as blood, the solubility of uric acid is low, and the imbalances between production and dissociation of uric acid can result in the buildup of excessive concentrations of uric acid in the blood, which could result in gout and kidney disorders [2]. In this regard, the Uox from external sources is commonly used to regulate the imbalances of uric acid levels in the blood [3,4]. In addition, the purified Uox enzyme is used as a diagnostic agent for determining the levels of uric acid in biological samples [5,6]. The *Aspergillus flavus*’ Uox, a homotetrameric enzyme, is the predominant form of this enzyme and is one of the most active Uox enzymes. Each subunit comprises a single polypeptide chain with a molecular weight of 34 kDa. The Uox enzyme encompasses four active sites, which are located between these subunits [7,8]. Moreover, it has been shown that the Uox enzyme does not require any cofactors or metal ions for its function [9]. However, the *A. flavus* Uox enzyme is relatively unstable, especially at temperatures above 35 °C (optimum temperature is 28–30 °C). This facile inactivation at higher temperatures has been attributed to the weak interaction between the subunits of the Uox and, therefore, their dissociation at relatively elevated temperatures [10]. Another feature of this tetrameric enzyme is that its stability seems to behave in a concentration-dependent manner, as in higher concentrations, it shows more stability. It has been suggested that an increase in the enzyme concentration strengthens the interaction between Uox subunits, which in turn reduces its inactivation rate [11]. Based on this intrinsic instability of Uox, a significant amount of work has been accomplished in improving its stability, including the incorporation of inter/intrachain disulfide bonds, PEGylation, and protein engineering studies [12,13,14]. Bacterial adhesion on material surfaces can be reduced by different types of coatings. For example, a silica surface on which polyelectrolyte multilayers are formed, with various proteins being the terminating layer, has been used by Bohnic et al. as a material surface [15].

Nanotechnology, the manipulation of matter at the atomic and molecular scale to create materials with remarkably varied and new properties, is a rapidly expanding area of research with huge potential in many sectors, ranging from healthcare to construction and electronics [16,17]. In medicine, it promises to revolutionize drug delivery, gene therapy, and diagnostics, and many areas of research, development, and clinical application [18,19,20,21]. Enzyme immobilization is one of the well-known approaches for improving the desired properties of enzymes as attested to by various studies [22,23,24,25,26,27]. Among these, the porous structured nanomaterials appear to be a suitable option for this purpose due to their unique structures [28]. One of the new and interesting classes of hybrid nanoporous materials is metal–organic frameworks (MOFs), which have garnered a lot of attention for enzyme immobilization applications; MOFs are porous coordination polymers comprising metal ions coordinated to the organic ligands [29,30,31,32]. The unique properties of MOFs include a large surface area, adjustable pore sizes, and high thermal tolerance [28,33], features that have rendered MOFs a very attractive option for the immobilization of various enzymes [34]. Among them, nickel-based MOFs have gained more interest not only because they have been demonstrated to have great application prospects for supercapacitors, but also because they could be the template/precursor for unique metal oxide and carbon materials with a specific structure, especially the Ni_3_(BTC)_2_·12H_2_O (BTC = 1,3,5-benzene tricarboxylic acid) [35,36,37,38,39]. Fe_3_O_4_–Ni MOF composites were found to exhibit minimal toxicity toward healthy cells, confirming their potential as safe biomedical materials. For example, MIL-100(Fe) was shown to maintain viability ≥85.97% in both normal liver HL-7702 cells, with negligible LDH leakage and apoptotic signs, indicating excellent biocompatibility even at substantial concentrations [40]. Similarly, magnetic Fe_3_O_4_ nanoparticles—including variants like Fe_3_O_4_@Au—were categorized as grade 0–1 non-cytotoxic for L-929 fibroblasts, induced virtually no hemolysis (<5%), and exhibited a high LD_50_ (~8.4 g/kg) in animal models [41]. Also, research has shown that Ni-based MOFs exhibit strong biocompatibility with healthy cells [42,43]. These studies collectively demonstrate that Fe_3_O_4_–Ni MOF systems and related iron oxide composites cause little to no harm to healthy mammalian cells, reinforcing their suitability for therapeutic and diagnostic applications. Besides, many authors have mentioned the good interaction between Ni and His-tagged enzymes where synthesized NPs have a porous structure and there are Ni atoms in the surface and inside the pores; good coordination between Ni atoms and enzymes has been reported [44]. On the other hand, magnetic nanoparticles (NPs) are attractive materials for enzyme immobilization and other applications, and, in view of their superparamagnetism, they can be accessed by remote stimuli [45,46,47]. This strategy can be exploited to immobilize specific molecules wherein magnetic NPs have been used for the conjugation of enzymes because of their specific features [48,49]. Magnetic nanoparticles, due to their high surface area, large surface-to-volume ratio, and easy separation under external magnetic fields, are highly valued in enzyme immobilization [50].

Despite the significance of *Aspergillus flavus* Uox for hyperuricemia therapy, there is the paucity of adequate data on the effect of immobilization of Uox on the Ni-MOF support. Consequently, the successful immobilization of *A. flavus* Uox on a Ni-based magnetic MOF nanomaterial is deliberated in an unprecedented report pertaining to the preparation and functional characterization of a Uox enzyme immobilized on any MOF materials. For this purpose, a Ni-based magnetic MOF (NimMOF) core–shell nanomaterial was prepared. Then, the recombinant *A. flavus* Uox enzyme was immobilized on NimMOF materials (Uox@NimMOF), and the physicochemical and structural properties of both, the Uox and Uox@NimMOF were analyzed. In brief, the kinetic properties of these immobilized enzymes, optimum pH and temperature, pH stability, thermal inactivation and stability, kinetics, and thermodynamics parameters were evaluated.

## 2. Materials and Methods

### 2.1. Materials

Ferrous chloride tetrahydrate (FeCl_2_·4H_2_O; purity 99%), ferric chloride hexahydrate (FeCl_3_·6H_2_O; purity 99%), methanol (Purity 98%), sodium hydroxide (NaOH; 98%), thioglycolic acid (TGA; 98%), and nickel chloride (NiCl_2_·4H_2_O; purity 99%) were obtained from Merck corporation. Uric acid, boric acid, and pyromellitic acid (1,2,4,5-benzene tetracarboxylic acid, H_4_BTEC; 98%) were procured from the Sigma-Aldrich Company, Darmstadt, Germany. In experimental performance, 0.4 T (Tesla) neodymium magnets were used. Isopropyl β-d-1-thiogalactopyranoside (IPTG) and kanamycin were purchased from BioBasic Inc., Markham, ON, Canada. BCA protein assay kits were secured from Takara, Shiga, Japan. Nitrilotriacetic (Ni-NTA) chromatography columns were acquired from Invitrogen, while the pET28a^(+)^ vector was from Novagene, Madison, WI, USA. All other analytic grade chemicals and were bought from Merck (Kenilworth, NJ, USA).

### 2.2. Preparation of the Recombinant Uox

*A. flavus* Uox’s coding sequence was created and cloned into the pGEM-B1 plasmid by the Bioneer Company (Daejeon, South Korea). After synthesis of the *A. flavus* Uox gene, it was subcloned into the pET-28a^(+)^ expression vector, under the regulator of T7 promoter, flanked by NcoI and XhoI sites. The resulted construct (called pET-28a-Uox) was then transformed into *E. coli* BL21 (DE3). The coding sequence of the Uox was confirmed by DNA sequencing. For the production of recombinant Uox, the altered *E. coli* BL21 (DE3) cells were induced by IPTG at a final concentration of 1 mM at 37 °C for 5 h. The induced cells containing the Uox enzyme were collected and were resuspended in 50 mM Tris-HCl (pH 8.5) by sonication on ice; sonication cycles of 30 s (with 30 s intervals) were repeated until most *E. coli* cells were lysed. The cell lysate supernatant was amassed by centrifugation at 13,000 rpm at 4 °C for 20 min. Subsequently, the His-tagged Uox enzyme was purified via a Ni-NTA Sepharose metal affinity column according to the manufacturer’s directions at 4 °C. Briefly, the cell lysate was supplemented with 500 mM NaCl and was loaded on the Ni-NTA column that was pre-equilibrated with the protein binding buffer (50 mM Tris-HCl, 500 mM NaCl, pH 8.0). The column was washed several times using the same buffer until the absorbance reached the baseline. Then, the Uox enzyme was eluted in a protein binding buffer supplemented with 500 mM imidazole. The purity of the prepared Uox was assessed using a 12% SDS-PAGE, and the purified Uox enzyme was finally concentrated using an Amicon centrifugal ultrafiltration column (10 kDa cut-off) (Sigma-Aldrich Company, Darmstadt, Germany); protein concentrations were determined using a BCA protein determination kit.

### 2.3. Synthesis of Ni-Based Magnetic MOF (NimMOF) Nanomaterial

First, superparamagnetic Fe_3_O_4_ NPs were produced using a co-precipitation approach for the fabrication of the magnetic MOF (NimMOF) core–shell nanomaterial [51]. Briefly, under a N_2_ stream, 100 mL of FeCl_2_ aqueous solution (0.5 M) was poured to the same volume of 1.0 M FeCl_3_. After stirring for around 40 min, 50 mL of sodium hydroxide (6.0 M) was added drop by drop to the prepared solution, and the mixture was rapidly agitated for about 2 h at 80 °C until a dark/black precipitate appeared. Fe_3_O_4_ NPs were then gathered using an external magnetic field and rinsed repeatedly with H_2_O. Finally, the magnetic Fe_3_O_4_ particles were accumulated and dried at 65 °C in an oven. In the next stage, a layer-to-layer synthesis technique was employed to create the magnetic MOF core–shell nanoparticles [52,53]. In a typical procedure, 300 mg of Fe_3_O_4_ NPs were mixed vigorously for five hours at room temperature with 15 mL of thioglycolic acid ethanolic solution (0.6 mM). After being surface modified with thioglycolic acid, the Fe_3_O_4_ NPs were captured using an external magnetic field and washed repeatedly with distilled water and ethanol, respectively. Next, 200 mg of functionalized Fe_3_O_4_ NPs were poured into 20 mL of NiCl_2_ ethanolic solution (10 mM) and stirred under ultrasound for 20 min until a homogenous solution was achieved. The precipitations were then collected using an external magnetic field and treated three times with ethanol. Thereafter, the precipitates were mixed in a 25 mL ethanolic solution of 1,2,4,5 benzenetetracarboxylic acids (10 mM) at 70 °C for 30 min. Using a magnetic field, the magnetic NPs were retrieved and washed with ethanol. After 20 cycles of adding NiCl_2_ and 1,2,4,5 benzene tetracarboxylic acid solutions, the final NimMOF core–shell nanomaterials were produced. Finally, NimMOFs were washed with ethanol and collected using a strong magnet and dried for 8 h under vacuum at 70 °C.

### 2.4. Characterization of the Synthesized NimMOF

FTIR spectroscopy of synthesized NimMOF and Fe_3_O_4_ NPs was performed by a Fourier transform spectrometer, Bruker TENSOR 27, Billerica, MA, USA. Moreover, the crystalline structure of NimMOF and Fe_3_O_4_ NPs was assayed by the XRD technique (Bruker Advance-D8 instrument and CuKα). The SEM method (FEI Quanta 200, Hillsboro, OR, USA) was used for the morphology observation of synthesized NPs, which operated at 25 kV.

### 2.5. Measurement of the Uox Activity

Uox activity was measured spectrophotometrically based on the established procedures [54]. Uox converts uric acid to Allantoin, which is monitored by observing a decrease in 293 nm adsorption; the measurement of the Uox activity is based on this reduction in absorbance. The assay reactions were carried out at room temperature (25 °C), and each reaction mixture (717 µL) consisted of boric acid buffer (20 mM, pH 8.5) and uric acid (48 µM). After adding 40 µL of the Uox enzyme to the reaction, any change in the absorbance of uric acid (293 nm) was recorded for 5 min.

### 2.6. Immobilization of Uox on NimMOF

For the preparation of Uox@NimMOF (i.e., Uox immobilized on NimMOF material), the synthesized NimMOFs in the previous steps were rinsed three times with a Tris-HCl buffer (50 mM; pH 8.0). Then, 200 μL of the enzyme solution (150 µg/mL) was added to 0.5 mg NimMOF of nanoparticles, and this mixture was stored at varying temperatures (4 and 25 °C). Afterward, nanoparticles were collected at 0, 15, 30, 45, and 60 min (with 15-min intervals) post-incubation using a magnet and washed three times with a 50 mM Tris-HCl buffer. At different time points, samples were taken. The activity of the remaining Uox in the supernatant and MOF pellet was measured for each sample.

### 2.7. Analysis of the Optimum Temperature for Uox Activity

The optimum temperatures of both, the naked and immobilized Uox (Uox@NimMOF) were determined from 10 to 70 °C (with 5 °C intervals) based on the Uox activity assay, as described in the previous sections. For these measurements, the substrate solution containing 20 mM boric acid (pH 8.5) and 48 µM uric acid were pre-heated for approximately 5 min at various temperatures. Then, the enzymatic solution was added to this pre-equilibrated reaction solution for performing the activity assays.

### 2.8. Thermal Inactivation and Thermal Stability Studies

Thermal inactivation studies were performed to evaluate the stability of the enzymatic function of both Uox and Uox@NimMOF at elevated temperatures. Therefore, both the control enzyme (138 µg/mL Uox) and Uox@NimMOF (containing 138 µg/mL of protein) solutions were incubated at 35 to 55 °C (with 5 °C intervals) for 5 min. Afterward, the samples were placed on ice for 2 min for a quick cool down and were then deployed in a Uox activity assay as described before. The Uox activity was considered 100% before the heat treatment. Furthermore, in the thermal inactivation studies, for the calculation of the inactivation rate constant (k_inact_) and half-life (T1/2) of both free Uox and Uox@NimMOF, first the graphs of the linear natural logarithm (Napier) based on the remaining activity of the enzyme were drawn and the k_inact_ value was obtained by dividing the half-life enzyme upon the Ln2 (0.693) [55,56]. Thermal stability studies were conducted at 45 °C for both the naked and immobilized enzymes. The enzyme solutions (with the same concentration) were incubated at 45 °C, and the activity of the treated enzyme was assayed every 5 min after holding the sample on ice for 2 min. Finally, the percentage of the relative activity was obtained. In addition, for the investigation of the long-term stability of the MOF-immobilized enzyme, both Uox and Uox@NimMOF samples were stored at 4 °C for approximately 2 months. Small samples were taken at different time points post-incubation, and their enzymatic performances were assessed according to the method described for measuring Uox activity.

### 2.9. Analysis of Kinetics and Thermodynamic Parameters

For the measurement of the kinetic parameters of Uox and Uox@mMOF samples, their enzymatic activities were measured at 25 °C using various concentrations of uric acid (ranging from 8–600 µM) in a boric acid buffer (20 mM, pH 8.5). Km values of uric acid were estimated based on the Lineweaver–Burk plot after two repetitions of tests. The catalyst rate constant (K_cat_) depends on the Vmax values and is obtained by the division of the Vmax on Km. The activation energy (E_act_) value is related to the k_inact_ value and was determined by drawing the Arrhenius law diagram and using the below Equation (1).

Ln (K) = Ln(C)^−Ea/RT^ (1)

In the above equation, k represents the constant inactivation enzyme (min^−1^), R is the global constant non-activation of gases (8.31 J·mol^−1^·K), T is the desired temperature (Kelvin), and C shows the Arrhenius constant.

Moreover, the enthalpy of the transition state (ΔH) and entropy (ΔS) was obtained from the inactivation experiments using Equations (2) and (3).

ΔH = Ea^−RT^ (2)

ΔS = (ΔH − ΔG)/T(3)

ΔG = −RT ln ^(kh/kBT)^ (4)

Moreover, the free energy of inactivation (ΔG) was calculated by using Equation (4), where h is the Planck’s constant (6.6262 × 10^−34^ Js), kB is the Boltzmann’s constant (1.3806 × 10^−23^ J·K^−1^), and k (s^−1^).

### 2.10. The Optimum pH and pH Stability of Uox Preparations

The optimum pH for Uox@mMOF and Uox was examined using a mixture buffer (100 mM KH_2_PO_4_, 50 mM sodium acetate, 50 mM Tris-HCl, and 50 mM glycine) at pH ranges from 4 to 12. Furthermore, pH stability for both cases of the enzyme (immobilized and naked) was measured by adding 40 µL of the enzymatic solutions into 50 µL of the mix buffer (pH = 6 to 12). The samples were kept at room temperature for 10 min while the activity of the enzymes was evaluated.

### 2.11. Intrinsic and Extrinsic Fluorescent Measurements

Intrinsic fluorescence spectra of Uox@mMOF and Uox were scanned and compared using a Cary Eclipse fluorescence spectrophotometer device. A solution containing 200 µg/mL of urate oxidase was prepared as the control and excited at a wavelength of 295 nm. Then, 500 µg NimMOF including 260 µg/mL urate oxidase was used to measure the intrinsic fluorescence spectra of the immobilized enzyme. To assess the structural conformation of the enzyme, it was immobilized and its free mode fluorescence spectra collected by excitation at 295 nm when the samples were first heated from 25 to 85 °C and then cooled to the room temperature. The extrinsic fluorescence study was accomplished in the presence of 30 µM ANS at a final concentration at 25 °C. Then, 1 µM protein solutions (equivalent to 600 µg Uox@NimMOF) were added into the ANS solution, and the fluorescence spectra were collected at various time intervals (0, 15, 30, and 60 min) using an excitation wavelength of 350 nm.

## 3. Results and Discussion

### 3.1. Preparation of Recombinant Uox

**Figure 1:**
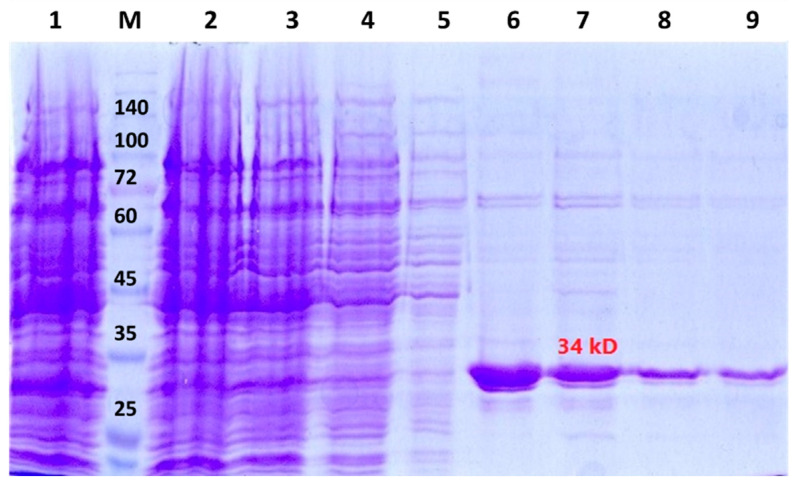
SDS-PAGE analysis of the purified recombinant Uox. Lane 1 shows the total proteins expressed in *E. coli*. Lane M is the standard weight protein marker. Lanes 2, 3, 4, and 5 represent the 1, 3, 6, and 14 washing samples. Lanes 6–9 show the eluted samples of the purified recombinant Uox.

Enzymes from various sources and with varying grades have been used for immobilization purposes. For example, when immobilized enzymes are destined to be used for the removal of pollutants in large environmental samples, crude enzymes are preferred due to economic reasons [57,58,59] Immobilized enzymes used for analytical and medical reasons generally need to be of higher purity [6,58,60]. In the current work, a purified recombinant Uox was used for the immobilization experiments. Accordingly, the *A. flavus* Uox gene was synthesized and placed into the expression vector pET-28a. *E. coli*, the most common expression system, was used for the recombinant production of the Uox enzyme, and the *A. flavus* Uox could be produced in an active and soluble form in the cytoplasmic space of *E. coli* [61,62] The recombinant Uox was expressed successfully in *E. coli* (Figure 1). Then, the soluble proteins were purified using Ni-NTA chromatography. The purity of the prepared Uox was ~95% as judged by the SDS-PAGE analysis (Figure 1). The purified Uox shows a molecular weight of around 34 kDa, which is consistent with the expected mass of the subunits of *A. flavus* Uox [7,63].

### 3.2. Characterization of the Magnetic MOF Nanoparticles (Ni-MOF)

Among different types of MOFs, the magnetic MOF (mMOF) nanomaterials offer some unique advantages to the immobilized enzymes [49]. These include the magnetic capture and distribution of the enzymatic preparations as well as improved stabilization [64]. The Ni-based core–shell mMOF nanomaterials used in this study were synthesized in a layer-by-layer approach [30]. The structural properties of these nanomaterials were investigated, and the results of these tests are provided in the following sections.

#### 3.2.1. FTIR Spectra of the Ni-MOF Particles

**Figure 2:**
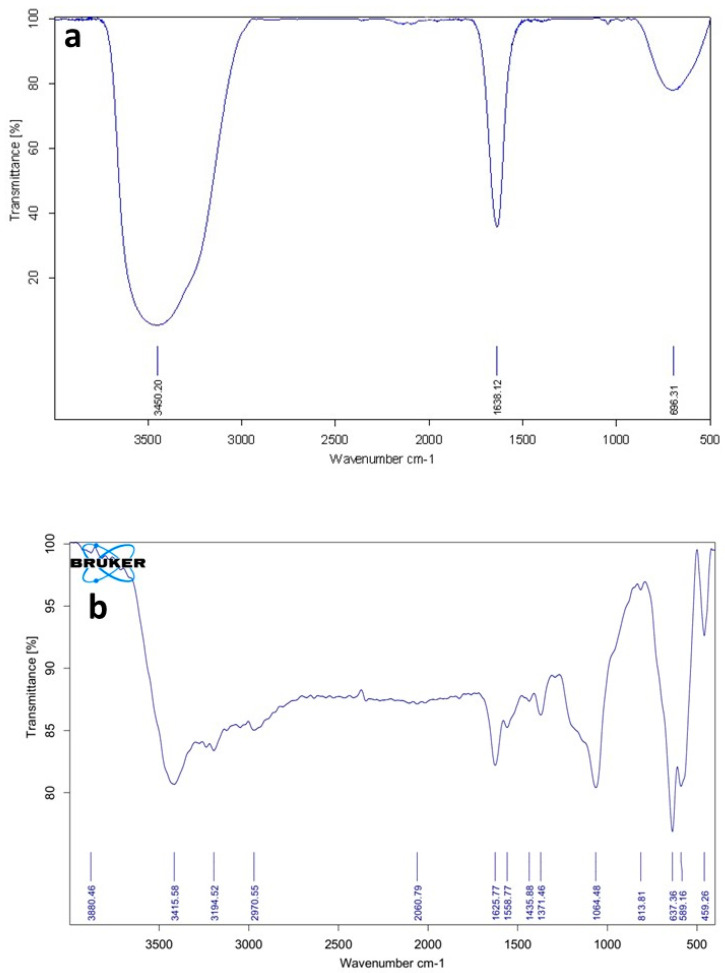
FTIR pattern of (**a**) Fe_3_O_4_ nanoparticles and (**b**) NimMOF particles.

The molecular structures of Fe_3_O_4_ nanoparticles as well as the NimMOF nanomaterials were first investigated by FTIR spectroscopy [30]. Figure 2a shows the FTIR spectrum obtained from the nanoparticles of Fe_3_O_4_. A sharp peak could be seen at the 693 cm^−1^ region in this spectrum; this can be ascribed to the M–O tetrahedral site in the spinel structure, which is more exposed in this sample. This could potentially be one of the explanations for the Fe_3_O_4_ particles’ fundamental character. The broad absorption band centered at 3450  cm^−1^ is attributable to the band O–H stretching vibrations, and the weak band near 1638 cm^−1^ is assigned to the H–O–H bending vibration mode due to the adsorption of water in air, as the FTIR sample disks were prepared in the open air.

Figure 2b represents a typical FTIR spectrum of the MOF nanocomposites used for the immobilization of Uox in this study. When porous structures of MOF were grown on the surface of Fe_3_O_4_ nanoparticles, two main peaks at 1558 and 1371 cm^−1^ could be observed. These peaks correspond to the asymmetrical and symmetrical COO vibrations, respectively. It can be proved that the Ni ions sufficiently coordinate the abundant amounts of carboxylate groups. Furthermore, a peak is evident at 1625 cm^−1^, which can be attributed to the C=O stretching vibration of the carboxylic groups of 1,2,4,5 benzene tetracarboxylic acids. The small peak observed at 813 cm^−1^ could be ascribed to the vibrations of Ni-O.

#### 3.2.2. XRD Patterns

**Figure 3:**
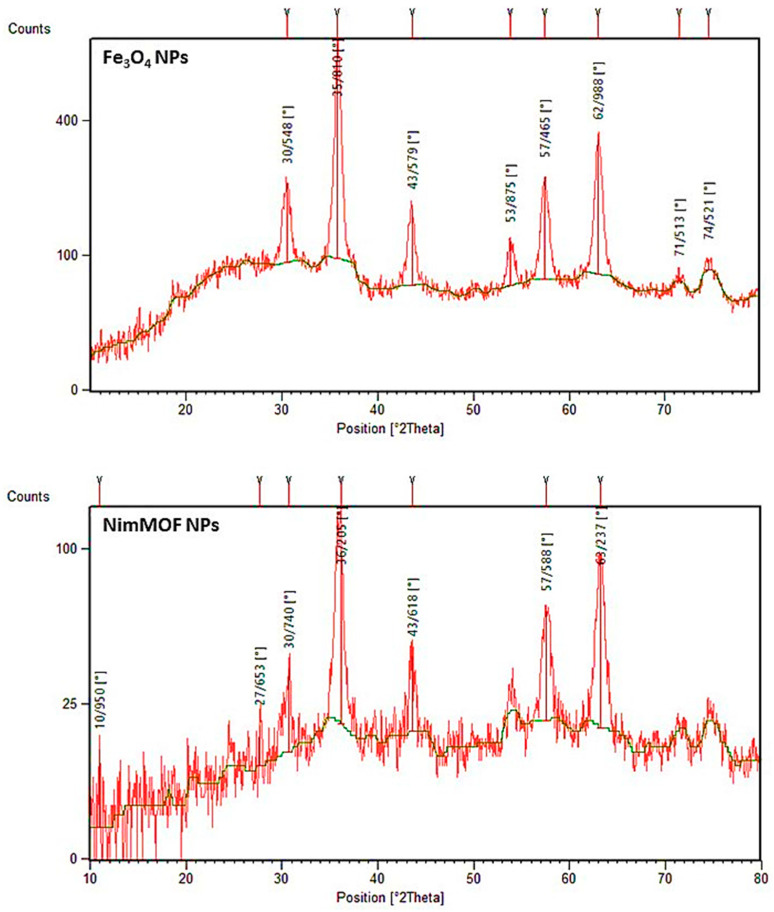
XRD pattern of the magnetic Fe_3_O_4_ and the NimMOF nanoparticles.

The XRD patterns of magnetic Fe_3_O_4_ NPs and NimMOF nanomaterials have spinal architectures and display seven distinct peaks at the area of 31.1°–75.8°, which correspond to different planes of the crystal lattice (Figure 3). Furthermore, the XRD results of NimMOF show that characteristic peaks do not shift after coating by MOF on Fe_3_O_4_ NPs (Xpert high score and Ref. code of 00–003-0875). The Debye–Scherrer equation was used to compute the crystallin sizes of Fe_3_O_4_ and NimMOF, which were determined to be about 16.6 and 56.3 nm, respectively. Furthermore, there were no intervening peaks in the NimMOF waveform. When porous structures were coated on the surface of magnetic Fe_3_O_4_ NPs, the XRD pattern demonstrates that crystallite size is indeed in nanometric levels, and the magnetic core composition is not affected.

#### 3.2.3. VSM and SEM Analysis of NimMOF

MOF materials are considered to have high dispersion and low density, and, therefore, the separation and handling of the enzymes that are immobilized on the MOF (i.e., enzyme@NimMOF) materials could be challenging. An innovative solution for this problem has been to use the MOF materials that have inherent magnetic properties (i.e., magnetic MOFs; NimMOFs). In particular, the engineered magnetic MOFs offer some valuable features, such as a large surface area, easy loading, and rapid collection [49]. This approach renders the NimMOFs particularly attractive for enzyme immobilization. These were the main factors involved in our preference for a magnetic MOF over the plain MOF for the immobilization of Uox.

**Figure 4:**
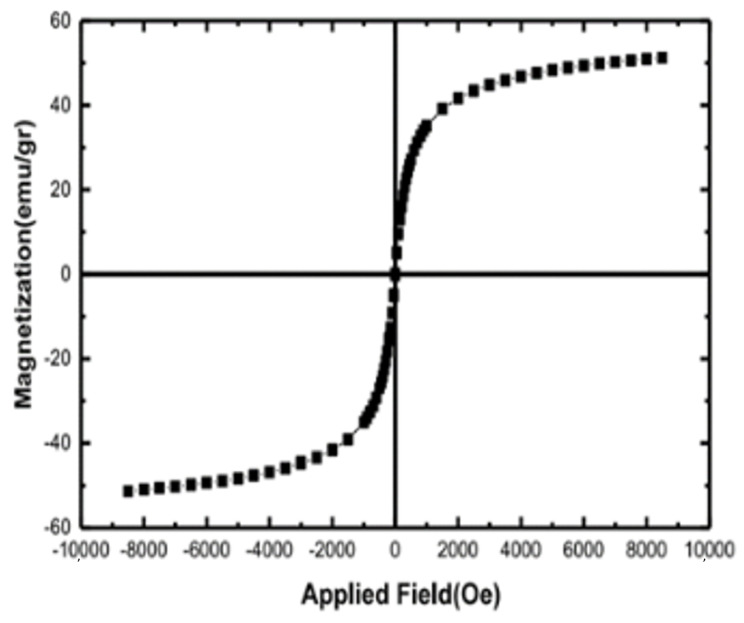
VSM analysis of the Ni-based mMOF nanomaterials.

After fabricating the Ni-based mMOF nanomaterials, their magnetic characteristics were investigated at 25 °C using a vibrating sample magnetometer (VSM) (Figure 4) [65]. The amount of saturation magnetization (MS) in the NimMOF was about 50.02 emu/g. At 25 °C, the magnetic hysteresis loops revealed that the NimMOF has no coercivity (Hc) or remanence (Br), thus revealing their superparamagnetic features; this superparamagnetism originates from the primary nanoparticles.

**Figure 5:**
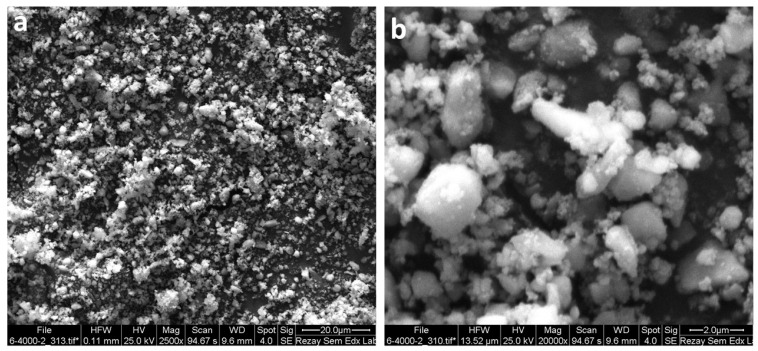
Scanning electron microscopy (SEM) images of the synthesized NimMOF at two different magnifications (**a**) and (**b**).

Figure 5a,b shows the scanning electron microscopy (SEM) images of the synthesized Ni-MOF at different magnifications. This figure shows the nanometric range and good distribution of the nanoparticles.

### 3.3. Analysis of the Performances of Uox and Uox@NimMOF

As stated earlier, the structural and physical properties of MOFs, which include an adjustable pore size, a large surface area, and high thermal stability, have made MOFs an attractive option for the immobilization of enzymes [28,33], and they have been used for immobilization of a set of diverse enzymes. Enzyme immobilization on MOF materials is completed through different processes. In the surface absorption process, which was conducted in this study for immobilizing Uox, weak interactions such as van der Waals and hydrogen bonds are involved. This method is the most common and effective way for immobilizing enzymes because it usually does not alter the structure of the enzyme and its active site [29]. The immobilization of Uox on the synthesized Ni-based mMOF nanomaterial is expected to occur by two mechanisms. First, the immobilization is likely to occur through the physical adsorption of Uox molecules in the NimMOF’s pore structures (i.e., on the surface of pore structures); such entrapment of enzymes is considered to be the main factor involved in the stabilization of the enzymatic performances [66]. Second, the existing Ni atoms (as a part of the mMOF) could coordinate with the available poly-histidine tags at the N-terminus of Uox molecules. Synthesized NPs have a porous structure and there are Ni atoms on the surface and inside the pores; reportedly, there is good coordination between Ni atoms and enzymes [44].Taken together, both of these proposed processes are capable of capturing the Uox enzyme, therefore improving its physical stability. Nonetheless, after immobilization of the Uox on the Ni-based mMOF nanomaterials, the behaviors of the enzymes had to be examined thoroughly to ensure a positive outcome. Therefore, the performances of both enzyme preparations (i.e., free Uox and Uox@NimMOF) under different conditions were tested and compared.

#### 3.3.1. The Optimum Temperature for the Uox and Uox@NimMOF Activity

**Figure 6:**
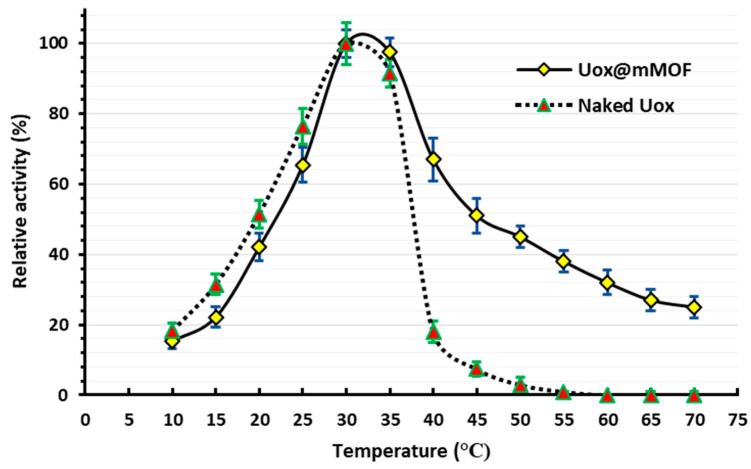
The optimum temperature of both Uox and Uox@NimMOF.

The activity of both the Uox and Uox@NimMOF was measured at various temperatures; Figure 6 shows that the maximum activity for both Uox and Uox@NimMOF is at approximately 30 °C. This result concurs with the previous studies, which have shown that the optimum temperature for *A. flavus* Uox enzyme is in the range of 28–32 °C [61,67,68].

#### 3.3.2. Thermal Inactivation and Thermal Stability Studies

Native *A. flavus* Uox is a tetrameric enzyme with relatively poor thermal stability, which has been attributed to the weak interaction between its subunits [10]. It has been suggested that at elevated temperatures (i.e., around and above 40 °C), the tetrameric Uox enzyme tends to disassociate into the inactive monomeric subunits [12]. For example, it was shown that at 35 °C, the Uox could lose its activity to below 20% of its original activity level, and also with any further increase in temperature, the Uox activity may decline further [69]. In addition, this inactivation has also been shown to happen in a concentration-dependent manner. For instance, Conley et al. showed that at 45 °C, the enzymatic solution with lower concentration was inactivated more readily than the higher concentration ones [11].

**Figure 7:**
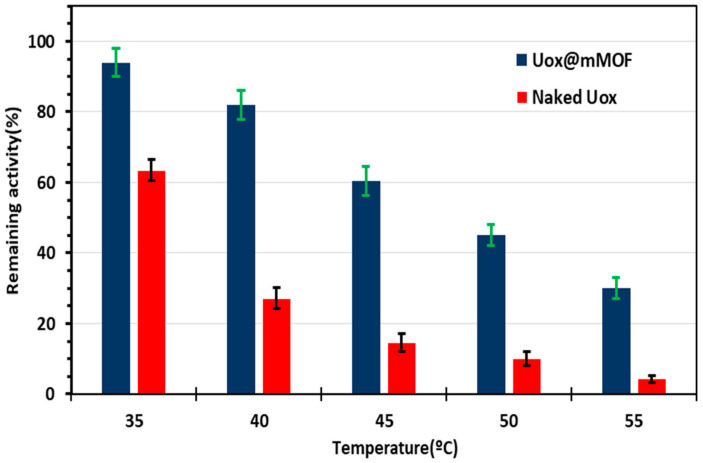
The remaining activity of Uox and Uox@NimMOF after heat treatment. Thermal inactivation of Uox and Uox@NimMOF at 45, 50, and 55 °C (for 5 min) is shown. Please note that the activity of Uox and Uox@NimMOF before the heat treatment was considered to be 100%.

While investigating the optimal temperatures for the function of both the free Uox and Uox@NimMOF preparations, as expected, a sharp and constant decrease in the activity of free Uox was observed as the temperature rose above ~30 °C, which was strictly consistent with the previously documented observations [10,68]. However, it was noticed that the process of immobilization of the Uox enzyme on the NimMOF nanomaterials (Uox@NimMOF) dramatically improved the thermal stability of the Uox (Figure 7). As it can be seen, while the free Uox enzyme lost almost all of its activity at ~40–45 °C, the immobilized Uox (Uox@NimMOF) retained around 60% of its original activity at 45 °C. Even at 70 °C, the highest temperature tested in our experiments, Uox@NimMOF still retained significant activity. The entrapment of Uox molecules in NimMOF pore structures (through surface absorption and/or by coordination of the terminal His-tag of Uox by the NimMOF’s Ni atoms) is responsible as the main factor involved in this improved physical stability.

In addition to assessing the details of Uox and Uox@NimMOF thermal inactivation, further tests were performed. Here, both samples (free Uox and Uox@NimMOF) were incubated at different temperatures (from 35 to 55 °C with 5 °C intervals) for 5 min. Then, the remaining activity of these heat-treated samples was measured (Figure 7). As it can be seen, the residual activity for the Uox@NimMOF sample was around 60, 45, and 30%, when incubated at 45, 50, and 55 °C, respectively. However, for the free Uox sample, the remaining activity was around 15, 10, and 4% at 45, 50, and 55 °C, respectively. This inactivation could be due to the disassociation of subunits of the Uox enzyme or denaturation of the enzyme and ultimately the loss of the active sites.

To better understand the effect of the NimMOF immobilization on the thermal stability of the Uox, the inactivation rate constant (k_inact_) for both the Uox and Uox@NimMOF was calculated. As presented in Table 1, the k_inact_ of the immobilized enzyme decreased, which is indicative of the effect of NimMOF in reducing the inactivation of Uox at elevated temperatures. Furthermore, half-life (T_1**/**2_) calculations show that the immobilization with NimMOF increased the enzyme’s half-life significantly (Table 1). Taken together, it is apparent that the NimMOF has a protective effect on the function of the Uox enzyme at higher temperatures.

**Figure 8:**
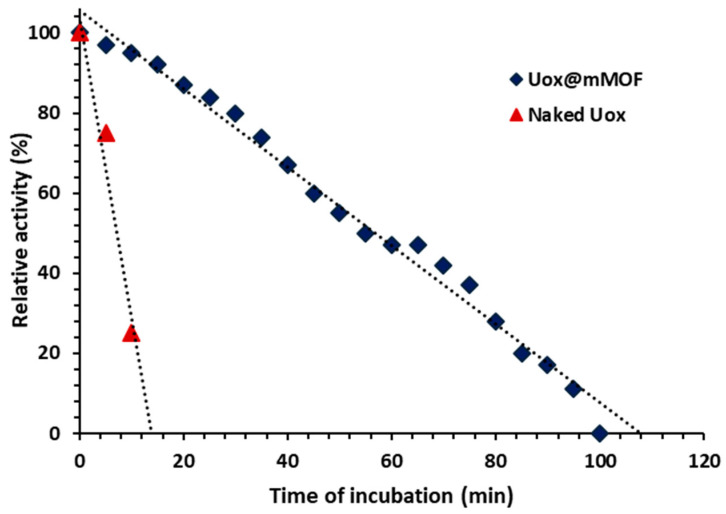
Thermal stability studies of Uox and Uox@mMOF.

For investigating the thermal stability of the enzyme, the activity of the samples treated at 45 °C was recorded every 5 min. The results indicate that the connections between NimMOF nanoparticles and enzymes probably have an important role in the thermal stability of the enzyme. Therefore, after 70 minutes of temperature treatment, Uox@NimMOF showed 20% activity (Figure 8).

#### 3.3.3. Analysis of Enzyme Kinetic and Thermodynamics Parameters

To further compare the free Uox and Uox@NimMOF, the kinetic parameters for these samples were calculated. These parameters were obtained over a range of substrate concentrations (8–600 µM uric acid) and are summarized in Table 2. The value of Km measured for the Uox enzyme is 50 µM, which is comparable to the data obtained in the reported studies [13,70,71]. However, the Km value for Uox@NimMOF did not change considerably in comparison to that of free Uox, thus indicating that the immobilization did not significantly change the affinity of the enzyme for its substrate. Furthermore, comparisons revealed a slight decrease in the turnover number (Kcat) of the immobilized enzymes (Table 2). The Kcat of Uox@NimMOF is approximately 85% of the free Uox, which could be due to the immobilization enzyme on the NimMOF nanomaterials.

Activation energy (Ea), which is associated with k**_inact_**, was calculated for the Uox enzyme and Uox@NimMOF (Table 3) to be 99.80 and 58.81 kJ·mol^−1^, respectively. The value of Ea decreased to about one-half of the free Uox enzyme, which indicates that at a high temperature upon immobilization on NimMOF, a lower amount of energy is required for activation of Uox. The ΔH value is a criterion for non-covalent enzymes (van der Waals, hydrogen bond, and electrostatic), which are broken down in the formation of a transition state complex for non-digestion of the enzyme. The value of ΔH for the naked Uox is 97 kJ·mol^−1^, but after immobilization it fell to 56 kJ·mol^−1^. In addition, ΔG is also defined in terms of enthalpy and entropy. The ΔG value in the Uox@NimMOF enzyme decreased by about 5 kJ·mol^−1^ proportional to the naked Uox enzyme, according to the results presented in Table 3. The ∆S parameters of the enzyme decreased after immobilization. Therefore, it can be concluded from the results that the nanoparticle provides an enzyme’s structural maintenance.

#### 3.3.4. Optimum pH and pH Stability for Uox Preparations

**Figure 9:**
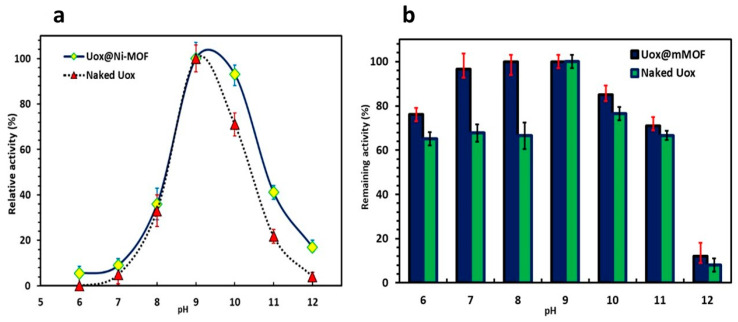
Analysis of the optimum pH and the pH stability of Uox preparations. (**a**) shows the level of activity of Uox and Uox@NimMOF samples at various pHs. (**b**) presents the pH stability results obtained for the free and immobilized Uox samples.

Optimum pH for the activity of both free Uox and Uox@NimMOF was measured and compared (Figure 9a). According to the results, the optimum pHs of 8.5–9.5 and 9–10 were obtained for free Uox and Uox@NimMOF, respectively, which lie well within the optimal pH ranges measured previously for the *A. flavus* Uox [61,67,68]. It is also worth mentioning that the activity of Uox@NimMOF in the pH ranging from 10 to 12 is approximately 20% more than that of the free Uox. This suggests that the Uox@NimMOF’s performance is higher than free Uox in a wider range of pH. In addition, Figure 9b shows the results obtained for the pH stability of the Uox samples. It can be seen that the immobilization of Uox on NimMOF material did not dramatically change the overall stability of this enzyme compared to the free enzyme in a wide range of pH; both Uox preparations were the most stable at pH ranging from 6 to 10. However, it should be mentioned that the immobilized Uox@NimMOF’s performance seems slightly superior to that of the free Uox in pH from 6 to 8.

#### 3.3.5. Storage Stability Studies

**Figure 10:**
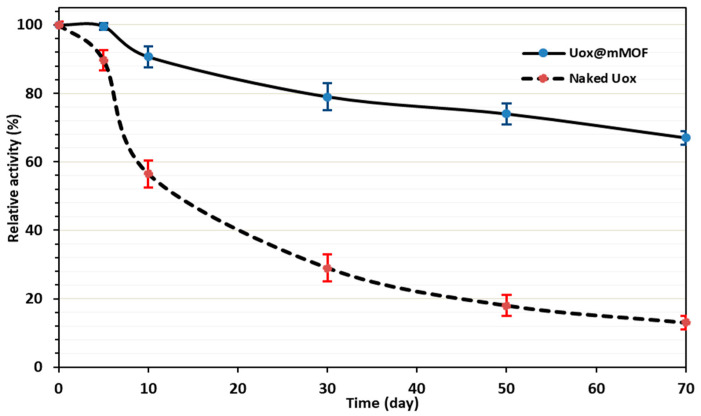
Storage stability analysis.

It was shown that the MOF immobilization could lead to the enhanced long-term stability of the encapsulated enzymes [66]. Thus, to assess the possible effects of immobilization on the long-term stability of Uox, both enzymatic solutions (i.e., free Uox and Uox@NimMOF) were stored at 4 °C for an extended period. Then, the activity for both samples was compared at different time points post-incubation at 4 °C, and the results are shown in Figure 10. It could be seen that immobilization of Uox on the NimMOF caused a significant increase in the stability of this enzyme when stored in solution at 4 °C for a prolonged period. After 50 days, while the activity of free Uox reached less than 20% of the original Uox preparation, the Uox@NimMOF sample still showed ~75% of its original activity. This improved long-term stability of the Uox enzyme could be attributed to its encapsulation in the Ni-based mMOF material.

#### 3.3.6. Florescent Measurements

**Figure 11:**
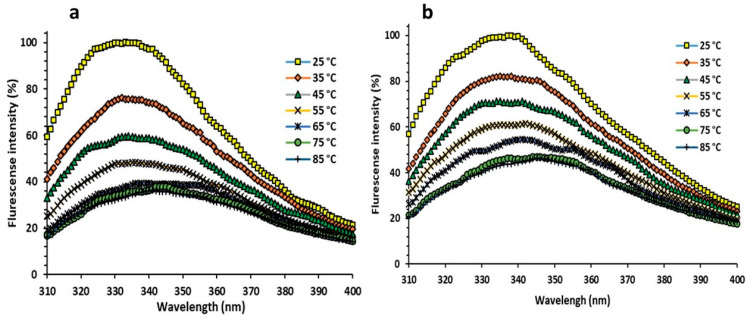
Internal fluorescence spectroscopy of naked and immobilized Uox. (**a**) shows the structural changes in the naked Uox against the increased temperature, and (**b**) shows the structural changes of the stabilized enzyme against the rise in temperature.

**Figure 12:**
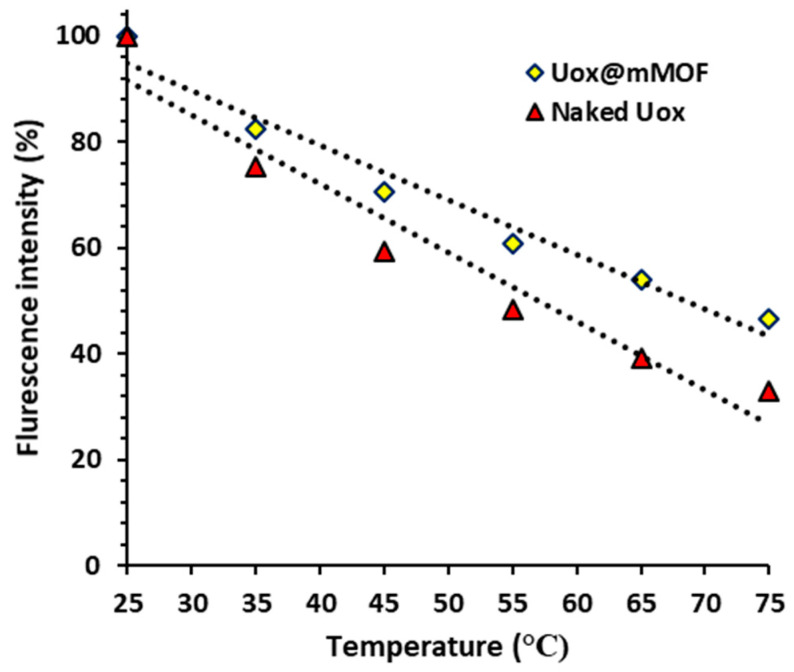
Shows the comparison of the structural changes of the stabilized enzyme (Uox@mMOF) and naked Uox against temperature variations.

Intrinsic fluorescence spectra of Uox and Uox@NimMOF were collected and compared at different temperatures. Considering that some of the proteins are removed from the surface of the particle by an increase in temperature, the released proteins were collected by centrifuge, and the structural changes of the immobilized urate oxidase were analyzed. As shown in Figure 11 and Figure 12, the intensity of the emitted light was considered 100% for both states (Uox and Uox@NimMOF) at 25 °C. As the temperature rises, the amount of fluorescence decreases with a different slope. Increased temperature causes structural changes and denaturation of proteins, but stabilized enzymes seem to maintain their structure better and reduce the slope of the fluorescent intensity more slowly.

**Figure 13:**
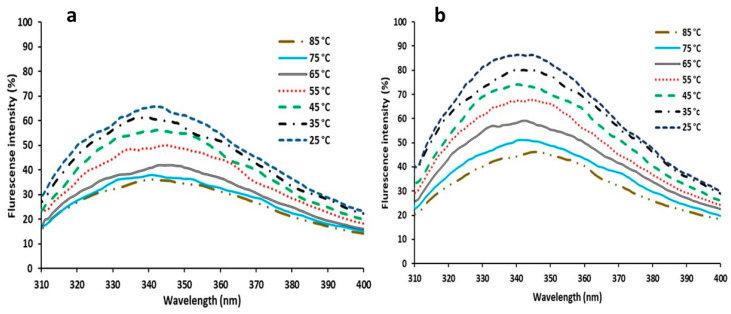
Internal fluorescence spectroscopy of naked and immobilized enzymes. (**a**) shows the extent to which the structure of the naked Uox can be retrieved. (**b**) also shows the return of the structure of the immobilized enzyme under the thermal reduction.

The renaturation of the protein structure was assessed after cooling the solution from 85 to 25 °C (Figure 13). By reducing the temperature, the percentage of fluorescent intensity was increased, indicating that the structures of the protein were refolded again.

**Figure 14:**
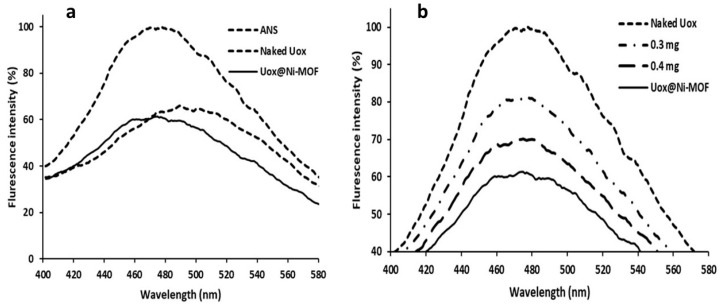
The hydrophobicity of the immobilized enzyme in the presence of ANS. (**a**) fluorescence intensity increased after the connection of ANS to hydrophobic regions, (**b**) depicts changes in the fluorescence intensity of the enzyme in the presence of different NimMOF concentrations.

Urate oxidase has hydrophobic patches on its surface [72,73], and it appears that its immobilization on the NimMOF nanoparticle can cover surface regions. For the confirmation of this thought, extrinsic fluorescence spectra of urate oxidase were investigated in both states (Uox and Uox@NimMOF) using 8-aniline-1-naphthalene sulfonic acid (ANS). As shown in Figure 14a, fluorescence intensity increased after the connection of ANS to hydrophobic regions and indicated the NimMOF could cover the hydrophobic areas that exist on the surface of urate oxidase. Figure 14b depicts changes in the fluorescence intensity of the enzyme in the presence of different NimMOF concentrations. It is also noteworthy to mention that the length of incubation time does not affect the output spectrum.

## 4. Conclusions

In conclusion, a significant amount of work was conducted on improving the stability of this enzyme, urate oxidase (Uox), including the use of additives such as an immobilized enzyme with alginate microcapsules that can degrade the increased uric acid in patients with renal insufficiency. Moreover, the binding of urate oxidase to albumin has caused thermal stability and resistance to proteolysis and increased the half-life of the enzyme to six times that of the wild type. Enzyme immobilization on the outer membrane of erythrocytes as a carrier can decompose uric acid with high efficiency. Conjugating urate oxidase with polyethylene glycol compounds or dextrin, as well as the use of nanoliposomal coating to prevent damage to the enzyme’s structure, improved the stability and activity of the enzyme. Furthermore, nanogels, methanol, dimethyl sulfoxide (DMSO; for strengthening the hydrogen bonds), glycerol, and NaCl have been deployed for the stabilization of this enzyme. In this study, we synthesized and used magnetized MOF (Ni-MOF) nanoparticles for the immobilization of the *A. flavus* Uox. Subsequent detailed investigations and comparisons of the properties of both the free Uox and the immobilized Uox (Uox@Ni-MOF) showed that a significant improvement in the thermal stability and long-term storage of Uox could be realized upon immobilization on Ni-MOF nanocomposite. Moreover, immobilization did not adversely affect the enzyme kinetic properties of Uox, as the immobilized enzyme remained active over a wide range of pH and temperatures. Furthermore, the ability to maintain the Uox enzymatic function is an important case that facilitates its application for commercial uses. This promising novel approach for immobilization of the Uox could potentially be used for the development of novel reagents for both therapeutic and laboratory purposes and especially for the construction of a uric acid assay kit with a longer shelf-life.

## Figures and Tables

**Table 1:** Thermal inactivation parameters for Uox and Uox@NimMOF.

	Temperature (K)	k_inact_ (min^−1^)	T_1/2_ (min)
Naked Uox	313	0.051	13.55
	318	0.067	10.23
	323	0.103	6.74
	328	0.313	2.21
Uox@NimMOF	313	0.019	36.55
	318	0.027	25.31
	323	0.035	19.58
	328	0.055	12.5

**Table 2:** Kinetics parameters of both Uox and Uox@NimMOF preparations.

	K_m_ (µM)	K_cat_ (min^−1^)
Naked UOX	50.5 ± 3.2	2.24 × 10^−6^
Uox@NimMOF	52.2 ± 4.1	1.92 × 10^−6^

**Table 3:** Thermodynamic parameters of Uox and Uox@NimMOF.

	T (°K)	Ea (KJ·mol^−1^)	∆H (KJ·mol^−1^)	∆G (KJ·mol^−1^)	∆S (KJ·mol^−1^ K)
Naked Uox	313	99.80	97.19	59.35	0.121
	318		97.16	61.06	0.113
	323		97.11	63.20	0.105
	328		97.07	67.26	0.091
Uox@NimMOF	313	58.81	56.20	56.76	−0.011
	318		56.16	58.68	−0.008
	323		56.11	60.34	−0.013
	328		56.07	62.54	−0.019

## Data Availability

Not applicable.
