# Correction: Motamedi et al. Enhancement of Thermostability of *Aspergillus flavus* Urate Oxidase by Immobilization on the Ni-Based Magnetic Metal–Organic Framework. *Nanomaterials* 2021, *11*, 1759

**DOI:** 10.3390/nano15181387

**Published:** 2025-09-10

**Authors:** Neda Motamedi, Mahmood Barani, Azadeh Lohrasbi-Nejad, Mojtaba Mortazavi, Ali Riahi-Medvar, Rajender S. Varma, Masoud Torkzadeh-Mahani

**Affiliations:** 1 Department of Biotechnology, Institute of Science, High Technology & Environmental Sciences, Graduate University of Advanced Technology, Kerman 7631133131, Iran; neda.m2950@yahoo.com (N.M.); mortezavimm@gmail.com (M.M.); 2 Medical Mycology and Bacteriology Research Center, Kerman University of Medical Sciences, Kerman 7616913555, Iran; mahmoodbarani7@gmail.com; 3 Department of Agricultural Biotechnology, Shahid Bahonar University of Kerman, Kerman 7616914111, Iran; azilohrasbi@gmail.com; 4 Department of Molecular and Cell Biology, Faculty of Basic Sciences, Kosar University of Bojnord, Bojnord 9415615458, Iran; riahi.ali@gmail.com; 5 Regional Centre of Advanced Technologies and Materials, Czech Advanced Technology and Research Institute, Palacký University in Olomouc, Šlechtitelů 27, 783 71 Olomouc, Czech Republic

## Text Correction

There was an update made to the original publication [1]. The authors have included information about cytotoxicity data from the literature in Section 1 Introduction, Paragraph 2:

Fe_3_O_4_–Ni MOF composites were found to exhibit minimal toxicity toward healthy cells, confirming their potential as safe biomedical materials. For example, MIL-100(Fe) was shown to maintain viability ≥85.97% in both normal liver HL-7702 cells, with negligible LDH leakage and apoptotic signs, indicating excellent biocompatibility even at substantial concentrations [40]. Similarly, magnetic Fe_3_O_4_ nanoparticles—including variants like Fe_3_O_4_@Au—were categorized as grade 0–1 non-cytotoxic for L-929 fibroblasts, induced virtually no hemolysis (<5%), and exhibited a high LD_50_ (~8.4 g/kg) in animal models [41]. Also, research has shown that Ni-based MOFs exhibit strong biocompatibility with healthy cells [42,43]. These studies collectively demonstrate that Fe_3_O_4_–Ni MOF systems and related iron oxide composites cause little to no harm to healthy mammalian cells, reinforcing their suitability for therapeutic and diagnostic applications.

There was an error in the original publication. The manufacturer and model of the SEM instrument have been updated in Section 2. Materials and Methods, 2.4. Characterization of the Synthesized NimMOF, Paragraph 1:

FTIR spectroscopy of synthesized NimMOF and Fe_3_O_4_ NPs was performed by a Fourier transform spectrometer, Bruker TENSOR 27, Billerica, MA, USA. Moreover, the crystalline structure of NimMOF and Fe_3_O_4_ NPs was assayed by the XRD technique (Bruker Advance-D8 instrument and CuKα). The SEM method (FEI Quanta 200, Hillsboro, OR, USA) was used for the morphology observation of synthesized NPs, which operated at 25 kV.

## Citation and References Correction

In the original publication [1], the following publications were not cited:

40. Chen, G.; Leng, X.; Luo, J.; You, L.; Qu, C.; Dong, X.; Huang, H.; Yin, X.; Ni, J. In Vitro Toxicity Study of a Porous Iron(III) Metal‒Organic Framework. *Molecules* **2019**, *24*, 1211. https://doi.org/10.3390/molecules24071211.
41. Chen, D.; Tang, Q.; Li, X.; Zhou, X.; Xue, W.; Zang, J.; Xiang, J.; Guo, C. Biocompatibility of magnetic Fe_3_O_4_ nanoparticles and their cytotoxic effect on MCF-7 cells. *Int. J. Nanomed.* **2012**, *7*, 4973–4982. https://doi.org/10.2147/ijn.s35140.
42. Rahimi, F.; Shahraki, S.; Hajinezhad, M.R.; Fathi-Karkan, S.; Mirinejad, S.; Sargazi, S.; Barani, M.; Saravani, R. Synthesis, characterization, and toxicity assessments of Silymarin-loaded Ni-Fe Metal-organic frameworks: Evidence from in vitro and in vivo evaluations. *J. Drug Deliv. Sci. Technol.* **2024**, *92*. https://doi.org/10.1016/j.jddst.2024.105372.
43. Alizadeh, N.; Salimi, A.; Hallaj, R.; Fathi, F.; Soleimani, F. Ni-hemin metal–organic framework with highly efficient peroxidase catalytic activity: toward colorimetric cancer cell detection and targeted therapeutics. *J. Nanobiotechnol.* **2018**, *16*, 1–14. https://doi.org/10.1186/s12951-018-0421-7.
48. Bilal, M.; Zhao, Y.; Rasheed, T.; Iqbal, H.M.N. Magnetic nanoparticles as versatile carriers for enzyme immobilization: A review. *Int. J. Biol. Macromol.* **2018**, *120*, 2530–2544. https://doi.org/10.1016/j.ijbiomac.2018.09.025.

These citations have now been inserted in 1: Introduction, Paragraph 2. 

There was an error in the original publication [1]. The following references have been removed:

29. Yang, M.; Li, C.; Zhang, Y.; Jia, D.; Zhang, X.; Hou, Y.; Li, R.; Wang, J. Maximum undeformed equivalent chip thickness for ductile-brittle transition of zirconia ceramics under different lubrication conditions. *Int. J. Mach. Tools Manuf.* **2017**, *122*, 55–65. https://doi.org/10.1016/j.ijmachtools.2017.06.003.
33. Zhao, N.; Deng, L.; Luo, D.; Zhang, P. One-step fabrication of biomass-derived hierarchically porous carbon/mno nanosheets composites for symmetric hybrid supercapacitor. *Appl. Surf. Sci.* **2020**, *526*, 146696. https://doi.org/10.1016/j.apsusc.2020.146696.
34. Chen, Z.; Zhang, H.; He, X.; Fan, G.; Li, X.; He, Z.; Wang, G.; Zhang, I. Fabrication of cellulosic paper containing zeolitic imidazolate framework and its application in removal of anionic dye from aqueous solution. *Bioresources* **2021**, *16*, 2644–2654. https://doi.org/10.15376/biores.16.2.2644-2654
47. Zhang, Y.; Li, C.; Jia, D.; Zhang, D.; Zhang, X. Experimental evaluation of MoS_2_ nanoparticles in jet mql grinding with different types of vegetable oil as base oil. *J. Clean. Prod.* **2015**, *87*, 930–940. https://doi.org/10.1016/j.jclepro.2014.10.027.
48. Zhang, H.; Guan, W.; Zhang, L.; Guan, X.; Wang, S. Degradation of an organic dye by bisulfite catalytically activated with iron manganese oxides: The role of superoxide radicals. *ACS Omega* **2020**, *5*, 18007–18012. https://dx.doi.org/10.1021/acsomega.0c01257?ref=pdf
49. Han, Y.; Li, H.; Feng, H.; Tian, Y.; Jiang, Z.; He, T. Mechanism of dislocation evolution during plastic deformation of nitrogen-doped Cocrfemnni high-entropy alloy. *Mater. Sci. Eng. A* **2021**, *814*, 141235. https://doi.org/10.1016/j.msea.2021.141235.
63. Wang, Q.; Sun, S.; Zhang, X.; Liu, H.; Sun, B.; Guo, S. Influence of air oxidative and non-oxidative torrefaction on the chemical properties of corn stalk. *Bioresour. Technol.* **2021**, *332*, 125120. https://doi.org/10.1016/j.biortech.2021.125120.
66. Zhang, K.; Yang, Z.; Mao, X.; Chen, X.-L.; Li, H.-H.; Wang, Y.-Y. Multifunctional textiles/metal-organic frameworks composites for efficient ultraviolet radiation blocking and noise reduction. *ACS Appl. Mater. Interfaces* **2020**, *12*, 55316–55323. https://doi.org/10.1021/acsami.0c18147
67. Zhang, K.; Huo, Q.; Zhou, Y.-Y.; Wang, H.-H.; Li, G.-P.; Wang, Y.-W.; Wang, Y.-Y. Textiles/metal–organic frameworks composites as flexible air filters for efficient particulate matter removal. *ACS Appl. Mater. Interfaces* **2019**, *11*, 17368–17374. https://doi.org/10.1021/acsami.9b01734.

With this correction, the order of some references has been adjusted accordingly.

The authors state that the scientific conclusions are unaffected. The Academic Editor approved this correction. The original publication has also been updated.
